# Dynamic Brain Activation and Connectivity in Elite Golfers During Distinct Golf Swing Phases: An fMRI Study

**DOI:** 10.3390/brainsci15111215

**Published:** 2025-11-11

**Authors:** Xueyun Shao, Dongsheng Tang, Yulong Zhou, Xinyi Zhou, Shirui Zhao, Qiaoling Xu, Zhiqiang Zhu

**Affiliations:** 1 School of Sports, Shenzhen University, Shenzhen 518060, China; 2 Shenzhen Institute of Neuroscience, Shenzhen 518057, China; 3 School of Computer Science and Engineering, South China University of Technology, Guangzhou 510641, China; 4 State Key Laboratory of Cognitive Neuroscience and Learning, Beijing Normal University, Beijing 100875, China

**Keywords:** action-observation network, functional magnetic resonance imaging, video observation, golf swing phases, functional connectivity

## Abstract

**Background/Purpose:** Skilled motor performance depends on the action–observation networks (AONs), which supports the internal simulation of perceived movements. While expertise effects are well-documented in sports, neuroimaging evidence in golf is scarce, particularly on temporal dynamics across swing phases. This study examines how golf expertise modulates AON activation and functional connectivity during temporally distinct swing phases (pre-hitting vs. hitting) and assesses implications for predictive-coding models of motor skill. **Methods:** Fifty-seven participants (elite golfers: *n* = 28; controls: *n* = 29) underwent functional magnetic resonance imaging (fMRI) scanning while viewing golf swing videos segmented into pre-hitting and hitting phases. Data analysis employed generalized linear models (GLMs) with two-sample t-tests for group comparisons and generalized psychophysiological interaction (gPPI) to assess functional connectivity using GLM-identified activation clusters as seeds. **Results:** (1) Compared to controls, elite golfers showed stronger activation in right insula and posterior cingulate cortex during pre-hitting, and in right cerebellum and bilateral postcentral cortex during hitting phases. The hitting > pre-hitting contrast revealed enhanced bilateral postcentral gyrus activation in golfers. (2) gPPI analysis demonstrated significant group × phase interaction in functional connectivity between right postcentral gyrus and left precuneus. **Conclusions:** Elite golf expertise dynamically retunes AON across swing phases, shifting from anticipatory interoceptive processing to impact-centered sensorimotor–parietal circuitry. These findings refine predictive-coding models of motor skill and identify the postcentral–precuneus loop as a potential target for neurofeedback interventions aimed at optimizing golf performance.

## 1. Introduction

Skilled motor behavior relies on the ability to recognize and internally simulate others’ actions [1,2,3]. Action–observation paradigms consistently demonstrate that viewing familiar movements activates the action–observation network (AON), a distributed sensorimotor system encompassing the premotor cortex, inferior parietal lobule, primary somatosensory cortex, and cerebellum [4,5]. For instance, experienced soccer players show heightened AON activation when observing sport-specific videos compared to neutral footage, indicating that expertise modulates perceptual–motor coupling [6,7]. While this “expertise effect” is evident in various ball sports, systematic neuroimaging evidence in golf is limited, particularly for temporal dynamics across distinct action phases.

Complex motor skills unfold in temporal phases, each engaging specialized yet interconnected neural processes through dynamic predictive coding mechanisms [8,9]. During preparation phases, the brain generates predictive models about upcoming movements, primarily involving prefrontal and parietal planning networks [10,11]. As actions progress into execution phases, sensorimotor regions actively compare incoming sensory feedback with these predictions, generating prediction errors that continuously update internal models and guide motor adjustments [12,13]. This predictive coding framework operates throughout the entire action sequence, with preparation phases emphasizing forward-model generation and execution phases focusing on error-correction and model refinement. However, it remains unclear whether these differences manifest during passive observation—evoking visual motor imagery—especially in golf, where expertise may enhance the precision of predictive processes across distinct swing phases.

Golf requires coordinated neural systems for attention, motor planning, sensorimotor coordination, temporal estimation, and affective regulation [14,15]. A full swing includes preparatory and striking–follow-through phases, potentially recruiting distinct substrates. Prior MRI studies provide foundational insights: professional golfers display stronger connectivity between the left cerebellum and occipital, temporal, parietal, and bilateral frontal regions than novices [16]. Our earlier quantitative MRI work also identified microstructural changes in the white matter of left temporal pole among elite golfers [17], suggesting that long-term training may induce localized myeloarchitectonic plasticity. Yet, these findings do not address neural dynamics during observation of golf swings, leaving two questions unresolved: (1) Do elite golfers engage the AON more robustly than controls during passive swing observation, isolating perceptual prediction from motor execution? (2) Are expertise-related neural differences phase-specific, given the swing’s temporal structure?

To address these gaps, we used an event-design functional MRI paradigm where elite golfers and matched controls viewed segmented golf swing videos (preparation vs. striking–follow-through phases). This design allowed us to isolate temporal components of motor observation and assess how neural processing diverges as a function of motor expertise. We hypothesized that golfers would exhibit stronger AON activation and connectivity in sensorimotor regions compared to controls, with the greatest differences during the striking phase. The significance of this research lies in its potential to refine current models of sensorimotor learning by demonstrating that motor expertise is not statically encoded but dynamically expressed across different action phases. By incorporating both activation and connectivity analyses, it could reveal how elite performers adaptively reconfigure internal models based on the anticipatory or executional demands of observed actions. Such findings have implications not only for the neuroscience of skill acquisition but also for designing targeted interventions (e.g., imagery training, neurofeedback) to optimize motor learning.

## 2. Methods

### 2.1. Participants

A total of 57 participants were recruited and assigned to two groups based on golf proficiency: the elite golf group (*n* = 28, 8 females; mean age = 24.93 ± 5.39 years; average golf score: 77.68 strokes per 18 holes, range 71–85, recorded on a par-72 course). The control group (*n* = 29, 8 females; mean age = 24.56 ± 4.68 years) comprised individuals with no formal golf training and minimal lifetime exposure (i.e., fewer than three recreational experiences with golf). One female participant withdrew from the experiment due to the discovery of an arachnoid cyst in the parietal lobe of her brain. All participants had normal or corrected to normal vision and were physically healthy and free of neurological disease, head injury and psychiatric disorder. They were paid for their participation and gave informed consent prior to experiment. Ethical approval was obtained from the Shenzhen Institute of Neuroscience (Approval No.: SION0013).

### 2.2. Experimental Design

**Figure 1:**
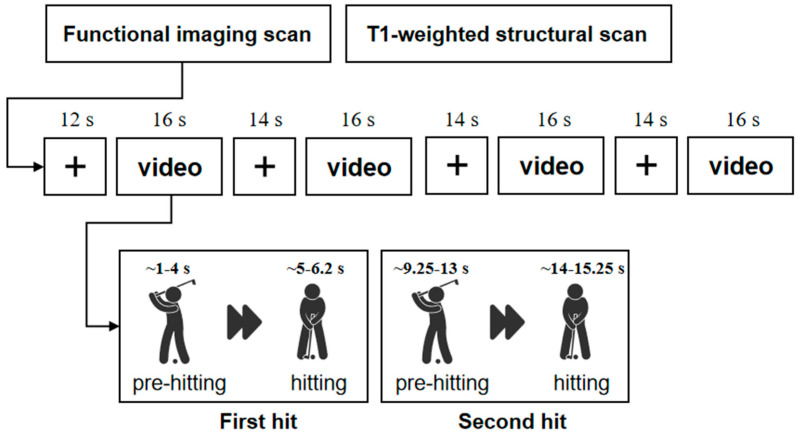
Experimental design.

All participants underwent MRI scanning, which included both T1-weighted structural imaging and functional imaging. During functional image acquisition, participants were instructed to focus their attention on a fixation point and watch golf swing videos presented on a screen. Specifically, each participant viewed four 16 s videos of golf swings, with each video containing two complete swing sequences. Each golf swing was clearly divided into the pre-swing preparation phase and the execution phase. The pre-hitting phase was defined as the period from the initiation of preparation for the swing to the top of the backswing. The hitting phase was defined as the period from the onset of the downswing to the completion of the follow-through. Each video was separated by a 16 s rest interval. The detailed experimental procedure is illustrated in Figure 1.

### 2.3. Image Acquisition and Preprocessing

Both high-resolution 3D structural brain data and fMRI data were acquired on a 3T Siemens Prisma MR system, using a 64-channel phased-array head coil. fMRI data were acquired using a multiple slice T2^*^-weighted echo-planer imaging (EPI) sequence with following parameters: repetition time (TR) = 2000 ms, echo time (TE) = 30 ms, flip angle = 90°, field of view (FOV) = 224 mm × 224 mm, data matrix = 64 × 64, voxel size = 3.5 × 3.5 × 3.5 mm^3^, number of slices = 33. Three-dimensional structural brain images were acquired using a T1-weighted 3D magnetization-prepared rapid gradient-echo sequence containing following parameters: TR/TE = 2530 ms/2.98 ms, flip angle = 7°, data matrix = 224 × 256, FOV = 256 mm × 224 mm, voxel size = 0.5 × 0.5 × 1 mm^3^, number of slices = 192. fMRI data were preprocessed stepwise by DPARSF (DPARSFA V5.2, http://rfmri.org/DPARSF, accessed on 1 March 2025) [18]. First, the images were slice-time corrected, realigned to correct for participant motion, segmented by DARTEL, normalized to the Montreal Neurological Institute (MNI) space using the structure information from coregistration, and smoothed by DARTEL with a Gaussian kernel (4 mm full-width at half-maximum).

### 2.4. Generalized Linear Models (GLMs)

GLM analysis was carried out using SPM12 software package (https://www.fil.ion.ucl.ac.uk/spm/software/spm12/, accessed on 1 March 2025). We first divided each video into two parts: pre-hitting and hitting. GLMs on the individual level were conducted by modeling the event sequence of each part as a hemodynamic response function. In order to control the variance from head movement, we also included six realignment parameters as regressors in the model. We then tested specific effects by setting vectors of related contrasts (pre-hitting, hitting, hitting vs. pre-hitting) and applied them into estimation of parameters. On the group level, two-sample *t*-tests were performed using the contrast results of control and golf player groups obtained from individual-level analysis.

### 2.5. Generalized Psychophysiological Interaction (gPPI)

To assess how watching different parts of videos modulated the functional connectivity between blood-oxygen-level-dependent (BOLD) signals in the seed region and target region, we performed the gPPI analysis using the gPPI toolbox [19]. Given that the right postcentral gyrus plays a critical role in visual perception and motor control [20], we chose it as the seed region in the gPPI analysis. We created a spherical ROI mask based on right postcentral gyrus activated in the interaction between Group (golf vs. control) and video content (hitting vs. pre-hitting). The MNI of the mask’ center was based on the center MNI of the right postcentral gyrus and the radius is 4 mm. We then extracted the BOLD time-series data using the spherical mask. For each participant, a PPI model was built with regressors of (i) main effect of the seed region, (ii) main effect of the contrast (hitting vs. pre-hitting), (iii) interaction between seed region and the contrast. A two-sample *t*-test was then carried out to test the group effect of the functional connectivity between seed region and target region.

## 3. Results

### 3.1. Regions Activated Between Groups

**Figure 2:**
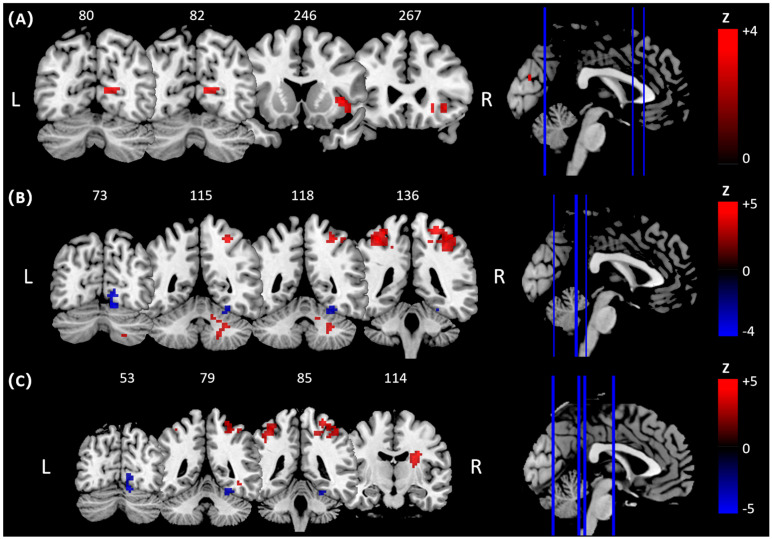
Brain activity for the contrast golf group > control group when watching golf playing videos at different temporal phases. (**A**) Pre-hitting phase. (**B**) Hitting phase. (**C**) Hitting phase > Pre-hitting phase contrast. Z values are indicated on the color bar. Activated brain regions with anatomical labeling and MNI coordinates are listed in Table 1. L, left; R, right.

In pre-hitting phase, whole-brain analysis during the pre-hitting (preparatory) phase (Figure 2A, Table 1-pre-hitting) revealed that, compared to the control group, the golf group showed significantly greater activation in the right insula (BA48, MNI: 39, 15, 0) and the right posterior cingulate cortex (PCC, BA17, MNI: 18, −60, 12). In the hitting phase (Figure 2B, Table 1-hitting), the golf group exhibited significantly increased activation in the right cerebellum (BA19, MNI: 18, −57, −27) and bilateral postcentral gyrus (left: BA2, MNI: −36, −39, 57; right: BA2, MNI: 27, −39, 66) relative to controls. Additionally, the right cerebellum (BA18, MNI: 12, −72, −15) showed greater deactivation in the golf group compared to the control group. In the direct contrast between the hitting and pre-hitting phases (Figure 2C, Table 1-hitting > pre-hitting), the golf group demonstrated significantly greater activation in the bilateral postcentral gyrus (left: BA2, MNI: −36, −39, 57; right: BA4, MNI: 33, −33, 66) compared to the control group.

### 3.2. The Difference Functional Connectivity in Conditions

**Figure 3:**
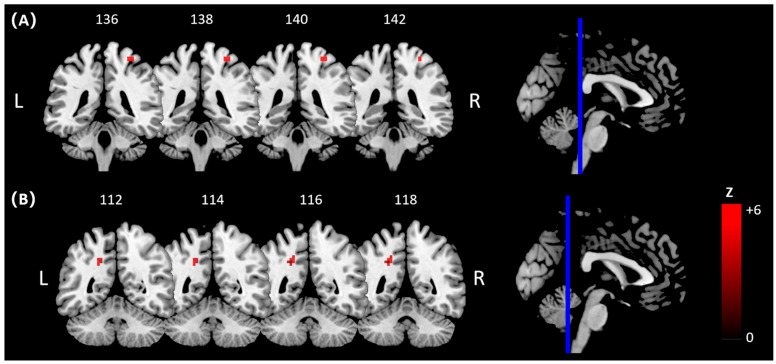
Functional connectivity between right postcentral gyrus and other brain areas in golf group compared with control group while watching golf playing videos. (**A**) A right postcentral gyrus mask based on activated results in the first-level GLM analysis. (**B**) Changes in connectivity based on right postcentral gyrus with left precuneus in the interaction between Video Content (hitting > pre-hitting) and group (golf group > control group).

With hitting or pre-hitting as the psychological context and the BOLD signal of the right postcentral gyrus as the physiological context (Figure 3A), psychophysiological interaction (PPI) analysis revealed an interaction between Group (golf vs. control) and Video Content (pre-hitting vs. hitting) in task-dependent connectivity of the right postcentral gyrus with left precuneus (Figure 3B; BA40, MNI: x = −21, y = −48, z = 36; Z = 5.09, *k* = 44).

## 4. Discussion

The present study demonstrates that elite golfers exhibit distinct phase-specific neural activation patterns during golf swing observation, with enhanced insula and posterior cingulate cortex activation during the pre-hitting phase and increased bilateral postcentral gyrus and cerebellar activation during golf swing hitting observation compared to controls. Critically, direct comparison between hitting and pre-hitting phases revealed that elite golfers showed preferential recruitment of bilateral postcentral gyrus during the striking phase, indicating phase-specific expertise effects in primary somatosensory processing. Furthermore, psychophysiological interaction analysis unveiled a significant group × phase interaction in FC between the right postcentral gyrus and left precuneus, demonstrating dynamic, context-dependent neural coupling associated with golf expertise. These findings provide novel evidence that AON are modulated by expertise in a temporally differentiated manner.

Consistent with our hypotheses and the broader action observation literature, elite golfers showed greater engagement of canonical AON regions compared to matched novices [21,22]. Crucially, our data advance this understanding by demonstrating a distinct temporal dissociation within the AON: during the preparatory phase, experts selectively recruited the anterior insula and posterior cingulate cortex—regions linked to predictive coding and interoceptive monitoring. This pattern suggests that elite performers enter an anticipatory mental state, simulating and evaluating the expected bodily outcomes of the impending stroke based on internal priors [23,24]. Notably, similar upregulation of the insula and PCC has been documented during the preparation period of other sports, such as basketball free-throws and fencing lunges, reinforcing the idea that high-level athletes employ a “prospective simulation” mode even during mere observation [23,25].

These phase-dependent neural activations suggest a dynamic engagement of predictive interoceptive (insula, PCC) and sensorimotor (postcentral gyrus, cerebellum) circuits across the swing, consistent with known AON and motor control frameworks. Critically, our passive observation paradigm is theoretically grounded in evidence that experts internally simulate observed sport-specific actions by recruiting the same motor representations engaged during actual execution [2]. This approach allowed rigorous temporal control while isolating perceptual–predictive mechanisms from motor–execution confounds, consistent with similar designs in basketball, soccer, and fencing research [7,23,25].

In contrast, during the execution phase, observation of the striking–follow-through period induced pronounced activation in bilateral postcentral gyrus and cerebellar lobules VI/VII among experts. While most previous studies have highlighted the roles of premotor and parietal cortices in action expertise, our results reveal that primary somatosensory and cerebellar regions—essential for efference-copy evaluation and precision control—are preferentially engaged during the most mechanically demanding segment of the swing [26,27]. The significantly greater activation of the postcentral gyrus during execution, relative to the preparatory phase, further emphasizes the temporal specificity of AON modulation in experts. Rather than a uniform upregulation, the expert network exhibits dynamic, phase-dependent reconfiguration throughout the observed action sequence—a process that may underlie the superior perception–action coupling and adaptability inherent to elite performance [23,28]. Nevertheless, it should be noted that fMRI, by its correlational nature, cannot establish whether the identified AON activations are causally involved in the learning of motor expertise or whether they represent experiential byproducts of extensive training. The present cross-sectional design thus precludes direct inferences about directionality. Future longitudinal or neurofeedback-based studies will be crucial to determine whether modulation of the postcentral–precuneus circuit actively contributes to the acquisition of motor expertise or merely reflects its consequence.

The PPI analysis shows that expertise reshapes sensorimotor communication as the observed action unfolds. A significant Group × Phase interaction emerged for FC between the right post-central gyrus (primary somatosensory cortex) and the left precuneus. Elite golfers strengthened this coupling during the hitting phase, whereas novices did not. Because the precuneus supports visuospatial imagery, embodiment and first-person perspective, its transient synchrony with primary somatosensory cortex likely binds fine tactile–proprioceptive predictions about club–ball impact to an egocentric body schema precisely when mechanical demands peak [29,30]. This short-lived “sensorimotor-to-self” link may enable rapid error monitoring and forward-model adjustment even during passive observation [31,32].

This connectivity finding resonates with and extends earlier resting-state work showing enhanced cerebello–parietal and premotor–precuneus coherence in expert marksmen, dancers and racing drivers [21,33]. Yet our data demonstrate that this reconfiguration is not static but dynamically gated by the temporal structure of the observed movement. Within a predictive-coding framework, the hitting segment carries the greatest kinematic uncertainty; allocating higher precision to somatosensory inputs and tightening their linkage with the precuneus therefore confers a clear advantage. Training protocols might exploit this window by combining impact-focused imagery or virtual-reality clips with haptic cues, thereby targeting the identified somatosensory–medial parietal loop [34,35]. Longitudinal work could test whether the emergence of phase-locked post-central–precuneus coupling marks the transition from novice to expert.

Although we primarily interpret the strengthened postcentral–precuneus connectivity as reflecting expertise-related sensorimotor coupling, it is also possible that this pattern partly reflects attentional or visuospatial processing demands. The precuneus has been implicated in spatial imagery, attentional orienting, and perspective taking [34]. Thus, the observed coupling might arise not solely from motor expertise but also from enhanced attentional engagement or visuospatial transformation during action observation. Future research could directly manipulate attentional load or visuospatial demands to disentangle these overlapping contributions.

Taken together, these findings provide direct evidence for dynamic, phase-dependent modulation of neural activity within the action observation network. By revealing that expertise-related neural coupling and regional engagement reconfigure across distinct swing phases, our study extends previous static accounts of motor expertise and highlights the temporally adaptive nature of sensorimotor processing in elite athletes.

Overall, our regional and network findings point to a temporally orchestrated redistribution of neural resources: anticipatory stages rely on interoceptive–evaluative hubs, whereas execution phases recruit a somatosensory–precuneus axis within the broader AON. From an applied perspective, these insights sharpen current models of perceptual-motor expertise and offer concrete neural targets for coaching and neurofeedback aimed at accelerating skill acquisition. By leveraging temporal windows of heightened neural receptivity, such as the hitting phase’s demand for sensorimotor precision, coaches and clinicians could develop neurofeedback- or imagery-based protocols to accelerate learning in novices. Moreover, the identified postcentral–precuneus loop may serve as a neural marker of training progression in future longitudinal research. While the present study employed a passive video viewing paradigm to ensure rigorous control over visual and temporal parameters, future research could incorporate motor imagery or combined action observation–imagery paradigms to further explore the generative processes underlying predictive motor simulation.

## 5. Limitations

This study has several limitations. First, the present findings are derived from a modest, demographically homogeneous sample of young Chinese athletes and controls; the limited statistical power and restricted age-cultural range caution against broad generalization to other populations or playing levels. Moreover, the control group consisted of golf-naïve participants to maximize the contrast in expertise-related neural dynamics; including amateur or novice golfers in future studies would help to capture intermediate stages of skill development. Second, because the study is cross-sectional, expertise-related differences cannot be disentangled from pre-existing neural traits or long-term training history; longitudinal or training-intervention designs are required to establish causality. Third, our paradigm relied on passive video observation without kinaesthetic feedback or actual club–ball impact, which may under-represent the multisensory and biomechanical demands of an authentic golf swing. Fourth, although task instructions emphasized attention to golf swing movements, we cannot fully exclude the influence of attentional strategy differences between groups. Future studies could explicitly manipulate attentional focus or ask participants to imagine the golfer’s sensations to further clarify this effect. Fifth, due to limited experimental resources, the present study did not include a unified measure of golf performance across participants. In addition, the elite golfer group was relatively homogeneous in both skill level and years of training, which restricted the feasibility of meaningful correlation analyses between neural activity and playing experience. Future studies incorporating objective behavioral indices and more heterogeneous athlete samples are needed to elucidate how expertise accumulation quantitatively modulates AON dynamics.

## 6. Conclusions

Elite golf expertise dynamically retunes the AON across swing phases, shifting from anticipatory interoceptive hubs to impact-centered sensorimotor–parietal circuitry. This phase-specific neural strategy refines predictive-coding models of skill and identifies actionable targets for imagery, neurofeedback and other interventions aimed at accelerating swing optimization in golf, particularly targeting the postcentral–precuneus loop.

## Figures and Tables

**Table 1:** Brain regions showing significant activation for the contrast golf group > control group during watching golf playing videos.

Comparisons		Brain Regions/BA	Peak MNI Coordinates			Cluster Voxels	Peak Z Values
			x	y	z		
Pre-hitting	Golf group > Control group	R Insula/BA 48	39	15	0	67	3.90 ^a^
		R Posterior Cingulate Cortex (PCC)/BA 17	18	−60	12	69	3.85 ^a^
Hitting	Golf group > Control group	R Cerebelum/BA 19	18	−57	−27	99	4.36 ^a^
		L Postcentral Gyrus/BA 2	−36	−39	57	105	4.26 ^a^
		R Postcentral Gyrus/BA 2	27	−39	66	255	4.04 ^a^
	Golf group < Control group	R Cerebellum/BA 18	12	−72	−15	114	4.05 ^a^
Hitting > Pre-hitting	Golf group < Control group	L Postcentral Gyrus/BA 2	−36	−39	57	175	4.54 ^a^
		R Postcentral Gyrus/BA 4	33	−33	66	199	4.06 ^a^

Note: The coordinates are from the Montreal Neurological Institute atlas. Significance was based on an uncorrected *p* value of 0.005 (*p* < 0.005), with a 50-voxel threshold. “^a^” indicates *P*_FWE_ < 0.05 (cluster-level). L, left.

## Data Availability

The raw data supporting the conclusions of this article will be made available by the authors on request.
